# A Study of Volatile Organic Compounds in Patients with Obstructive Sleep Apnea

**DOI:** 10.3390/metabo15010042

**Published:** 2025-01-11

**Authors:** Chuan Hao Gui, Zhunan Jia, Zihao Xing, Fuchang Zhang, Fang Du, Alex Chengyao Tham, Ming Yann Lim, Yaw Khian Chong, Agnes Si Qi Chew, Khai Beng Chong

**Affiliations:** 1 Tan Tock Seng Hospital, 11 Jln Tan Tock Seng, Singapore 308433, Singapore; chuanhaogui@gmail.com (C.H.G.);; 2 Breathonix Pte Ltd., Block 71 Ayer Rajah Crescent, #05-19/20/21, Singapore 139951, Singaporefuchang@breathonix.com (F.Z.);

**Keywords:** clinical diagnostics, PTR-MS, breath analysis, obstructive sleep apnea, biomarkers

## Abstract

**Background**: Obstructive Sleep Apnea (OSA) is a prevalent sleep disorder characterized by intermittent upper airway obstruction, leading to significant health consequences. Traditional diagnostic methods, such as polysomnography, are time-consuming and resource-intensive. **Objectives**: This study explores the potential of proton-transfer-reaction mass spectrometry (PTR-MS) in identifying volatile organic compound (VOC) biomarkers for the non-invasive detection of OSA. **Methods**: Breath samples from 89 participants, including 49 OSA patients and 40 controls, were analyzed using PTR-MS. Significance analysis was performed between OSA patients and controls to identify potential biomarkers for OSA. To as-sess the differences in VOC concentrations between OSA patients and control subjects, the Wilcoxon rank-sum test was employed. partial least squares discriminant analysis (PLS-DA) analysis and heatmap plot was conducted to visualize the differentiation between OSA patients and control subjects based on their VOC profiles.In order to further investigate the correlation between identified biomarkers and the severity of OSA measured by Apnea–Hypopnea Index (AHI), regression analysis was conducted between biomarkers and AHI Index. **Results**: The results identified specific VOCs, including m045 (acetaldehyde), m095.950, and m097.071, which showed significant differences between OSA patients and controls. Advanced statistical analyses, including PLS-DA and correlation mapping, highlighted the robustness of these biomarkers, with m045 (acetaldehyde) specifically emerging as a potential biomarker associated with the AHI Index. **Conclusions**: This study underscores the potential of VOCs as biomarkers for identifying patients with severe AHI levels. The analysis of VOCs using PTR-MS presents a rapid, non-invasive, and cost-effective method that could be seamlessly integrated into clinical practice, allowing clinicians to better stratify patients based on their need for polysomnography and prioritize those requiring earlier testing. Future studies are necessary to validate these findings in larger cohorts and to explore the integration of PTR-MS with other diagnostic modalities for improved accuracy and clinical utility.

## 1. Introduction

Obstructive Sleep Apnea (OSA) is a prevalent sleep disorder characterized by recurrent episodes of partial or complete upper airway obstruction during sleep, leading to intermittent hypoxia, sleep fragmentation, and daytime sleepiness [1,2]. It is associated with various risk factors, including obesity, craniofacial abnormalities, and advancing age [3,4,5]. The pathophysiology of OSA involves the collapse or narrowing of the upper airway, often exacerbated during sleep due to reduced muscle tone and altered respiratory control mechanisms [4]. Clinical manifestations of OSA range from snoring and witnessed apneas to systemic complications such as hypertension, cardiovascular disease, and cognitive impairment [6].

The accurate detection of OSA relies on a combination of screening questionnaires, nasoendoscopy, and an overnight sleep study. Although questionnaires like the Epworth Sleepiness Scale (ESS) and STOP-BANG criteria are widely accepted and validated screening tools [7], they have limitations, particularly in terms of lower specificity [8]. Polysomnography (PSG) remains the gold-standard diagnostic tool, allowing for a comprehensive assessment of respiratory parameters, sleep architecture, and associated abnormalities during overnight sleep monitoring [9]. While PSG is highly sensitive and specific for diagnosing OSA, it has limitations, such as cost, the availability of specialized sleep centers, and patient discomfort during overnight monitoring [9]. Home sleep apnea testing (HSAT) may be used as a more accessible and cost-effective alternative in certain cases, particularly for uncomplicated OSA presentations [10,11]. HSAT, although more convenient, may underestimate OSA severity in complex cases or fail to capture certain sleep-related parameters accurately [12].

Volatile organic compounds (VOCs) are a diverse group of organic chemicals emitted from various metabolic processes within the body and can be found in the breath [13,14,15]. These compounds, present in trace amounts, can serve as potential biomarkers for a range of diseases, including Obstructive Sleep Apnea (OSA). Biomarkers are measurable indicators of a biological condition or state, and in the context of breath analysis, they provide a non-invasive means of disease detection and monitoring. VOCs have been successfully used to detect a range of conditions [16], including cancer, metabolic disorders, and respiratory and gastrointestinal diseases, due to their ability to reflect underlying changes in metabolic and inflammatory processes.

Breath tests offer a compelling diagnostic tool due to their non-invasive nature, ease of sample collection, and the potential for rapid analysis [17,18,19,20,21]. The process involves collecting exhaled breath from patients, which is then analyzed using advanced techniques, such as proton-transfer-reaction mass time-of-flight spectrometry (PTR-TOF-MS). PTR-TOF-MS is a highly sensitive method that allows for the real-time detection and quantification of VOCs in breath samples, making it a valuable tool for identifying disease-specific biomarkers.

Breath analysis using PTR-TOF-MS and the identification of VOC biomarkers represent a promising frontier in medical diagnostics. This approach not only enhances our understanding of the metabolic changes associated with various diseases but also opens up new avenues for personalized medicine, where treatments can be tailored based on the specific VOC profile of an individual patient.

Given the complexity and high dimensionality of VOC data, multivariate analysis techniques such as PLS-DA, heatmaps, and correlation maps are essential for identifying patterns and relationships within the dataset [22]. PLS-DA helps reduce data dimensionality, allowing the visualization of group separations and identifying the VOCs contributing most significantly to differences between OSA patients and controls. Heatmaps and correlation maps further reveal specific clusters and associations, providing insights into the metabolic profiles of disease states. Together, these methods enhance the interpretability of VOC data and aid in pinpointing potential biomarkers, supporting the development of effective diagnostic tools.

The integration of VOCs into the diagnostic framework for OSA represents a significant advancement in early detection and risk stratification [23]. This study aims to evaluate the efficacy of breath analysis for detecting VOC biomarkers associated with OSA and to propose its use as an adjunct to current questionnaire-based screening tools [24,25]. By incorporating VOC breath analysis into current screening tools, we seek to enhance the stratification of patients based on their risk of OSA. This approach will enable more informed decisions regarding the need for hospital-based PSG or recommending patients for HSAT, ultimately optimizing resource allocation and improving patient outcomes.

## 2. Method

### 2.1. Study Population

This study involved a total of 89 participants, divided into two distinct groups: 49 patients with Obstructive Sleep Apnea (OSA) and 40 controls. All participants were recruited from Tan Tock Seng Hospital Singapore, a major healthcare institution renowned for its comprehensive medical services and research facilities, between May 2022 and June 2024 after obtaining informed consent.

All study participants had undergone formal Level 1 polysomnography and were invited to participate in this study during the review of their PSG results [26]. Participants with an AHI of 15 or lower were recruited into the control group, while participants with an AHI of more than 15 were recruited into the study group. Other relevant demographic data, medical history, and clinical parameters were also collected for this study. The exclusion criteria for this study include recent food or beverage consumption within the past hour or adherence to a ketogenic diet, alcohol intake within the past 6 h, a history of cancer, pregnancy, respiratory or heart failure, liver dysfunction or failure, renal failure, and uncontrolled diabetes mellitus. These exclusion criteria were selected based on the proposed framework for conducting and reporting studies on VOCs, with the aim of minimizing confounding factors, particularly those affecting metabolic processes or respiratory function, which could interfere with the detection of volatile organic compounds [27,28].

Relevant information about each participant was recorded, including demographic data, polysomnography results, and relevant past medical history.

The trial was approved by the NHG Domain Specific Review Board (DSRB) (Ref: 2019/00367), and all enrolled subjects gave signed informed consent prior to the study.

### 2.2. PTR-TOF-MS Measurement and Analysis

Breath samples were measured using a PTR-MS TOF1000 (Ionicon Analytik GmbH, Innsbruck, Austria). PTR-TOF-MS consists of an ionization section and a detection section [29]. During the ionization process, protonated water ions ($H_{3}O^{+}$) were generated through a hollow cathode discharge in the ion source. These $H_{3}O^{+}$ ions were subsequently introduced into the drift tube by an electric drift field, facilitating the chemical ionization of volatile organic compounds (VOCs) in breath samples via proton-transfer reactions (PTRs). Only VOCs with a higher proton affinity (PA) value than that of H2O molecules underwent ionization by $H_{3}O^{+}$ and proceeded to the detection section. The ionized VOCs were then directed by an electric field toward the time-of-flight mass spectrometer (TOF-MS), where they were differentiated and detected based on their mass-to-charge ratios (*m*/*z*). Because of this ionization method, the molecular weight after ionization is one unit mass greater than the molecular weight before ionization. By measuring the count rate of both reagent ions and product ions, real-time quantification is achieved. PTR-TOF-MS can detect compounds at parts per billion by volume or even parts per trillion levels in real time.

Key operational parameters of the PTR-MS instrument included a drift tube voltage of 600 V, a temperature of 80 °C, a drift tube pressure of 2.3 mbar, and an E/N ratio of 139 Td. Additionally, the sampling line and buffer tube were maintained at 70 °C. The sampling line and buffer tube were maintained at a temperature of at least 70 °C to minimize the condensation of water vapor and reduce the risk of contamination by viruses and other particulate matter. Elevated temperatures help to prevent the accumulation of moisture, which can act as a carrier for pathogens, thus improving the accuracy and safety of the sample collection process. Table 1 shows the key operational parameters for data collection using PTR-MS.

After participants registered their personal data and signed consent forms, they were instructed by a study team member to exhale into a gas sampling bag (Tedlar PVF film), which was then sealed and transported to the research laboratory on the same day. Before measurements, the device was calibrated. The bag was connected to the PTR-MS device and sealed using laboratory film (Parafilm). Initially, the PTR-MS was connected to the external environment until the pressure parameters stabilized. Once stable, the valve of the sampling bag was opened, and the device began drawing air from the bag. Observing the pressure parameters ensured they were stable before data collection commenced. This procedure minimizes pressure fluctuations in the drift tube, ensuring accurate readings [27,28].

The described procedure addresses several potential issues and streamlines the collection process. Starting measurements from normal atmospheric pressure avoids the lengthy stabilization period required if starting from lower pressures, which can sometimes result in the device reaching only half the target pressure and significantly skewing the data. Consequently, we avoid switching from standby mode to active measurement mode. Since the Tedlar bag valve is initially closed, if the bag is connected from the start, the lack of gas input prevents the PTR-MS pressure from rising until the valve is opened. This situation is akin to starting from standby mode, which we aim to avoid.

The PTR-MS calculates the concentration of all VOCs and saves the data in .h5 files [29]. These raw concentration data were processed using Viewer software 4.2.0 (Ionicon Analytik GmbH, Innsbruck, Austria) for mass calibration and peak data calculation. The software identified background air and exhaled breath, selecting and averaging data points from the end-tidal phase for the three exhalations from each subject. VOCs with concentrations lower in breath than in the background were excluded from subsequent data analysis. An Excel file containing the list of VOCs and their concentrations (in parts per billion, ppb) was generated for each sample.

## 3. Results

Data analysis was performed using R (4.3.0). Multiple dimensions of the data were analyzed, and significance analysis was performed to rank *m*/*z* values based on their *p*-values to identify potential biomarkers. The data were also visualized using PLS-DA plots, heatmaps, and correlation maps.

### 3.1. Study Characteristics

Table 2 shows the demographics and AHI values, comparing the OSA and control groups. The OSA group consists of 49 individuals with a mean age of 52.61 ± 11.36 years, while the control group includes 40 individuals with a mean age of 43.38 ± 12.70 years. The gender distribution in the OSA group is 67.3% male and 32.7% female, whereas the control group has a distribution of 60.0% male and 40.0% female. The AHI is markedly higher in the OSA group, with a mean value of 50.98 ± 26.05, compared to 6.68 ± 3.96 in the control group.

### 3.2. OSA vs. Control Analysis

#### 3.2.1. Significance Analysis

Significance analysis was performed to identify potential biomarkers for OSA. To assess the differences in VOC concentrations between OSA patients and control subjects, the Wilcoxon rank-sum test was employed. This non-parametric test is suitable for analyzing small datasets or cases when the assumption of normality is not met, providing a reliable method to compare two independent groups. Table 3 presents the biomarkers with their corresponding p-values. For instance, m063, m095.950, and m097.071 were identified as significant, with p-values of 0.042750951, 0.001048342, and 0.011163276, respectively. These markers suggest specific VOCs that are notably different in concentration between OSA patients and control subjects.

#### 3.2.2. Partial Least Squares Discriminant Analysis (PLS-DA)

**Figure 1:**
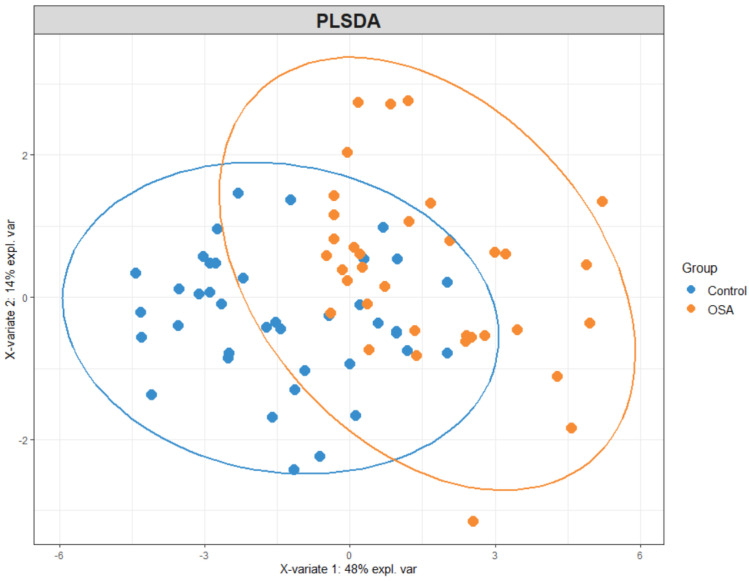
PLS−DA plot for OSA vs. control.

PLS-DA was conducted to visualize the differentiation between OSA patients and control subjects based on their VOC profiles. The PLS-DA plot (Figure 1) demonstrates a clear separation between the two groups, indicating distinct metabolic signatures. The first two principal components accounted for a significant portion of the variance, highlighting the effectiveness of VOCs in distinguishing OSA from controls.

#### 3.2.3. Heatmap Analysis

**Figure 2:**
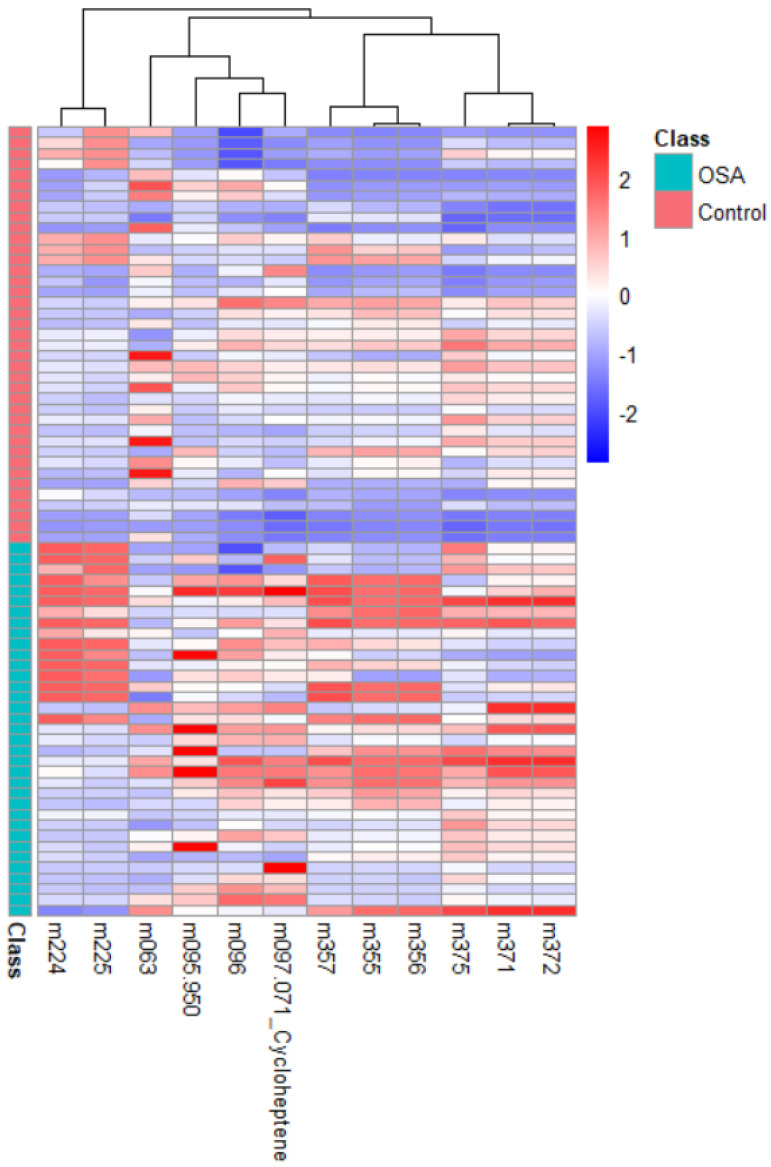
Heatmap plot for OSA vs. control.

The heatmap (Figure 2) presents the relative abundance of identified VOCs in both the OSA and control groups, revealing distinct clustering patterns based on VOC concentrations. In this visualization, the *X*-axis represents individual VOCs, while the *Y*-axis corresponds to each sample, either from OSA patients or controls. The color gradient, scaled from −2 to +2, indicates normalized VOC concentrations: positive values represent higher concentrations, while negative values reflect lower concentrations relative to the dataset’s mean. The heatmap shows certain VOCs with markedly elevated concentrations in the OSA group, underscoring their potential as biomarkers. This distinct clustering between OSA patients and controls further supports the differentiation capability of these VOCs in identifying OSA.

#### 3.2.4. Correlation Map

**Figure 3:**
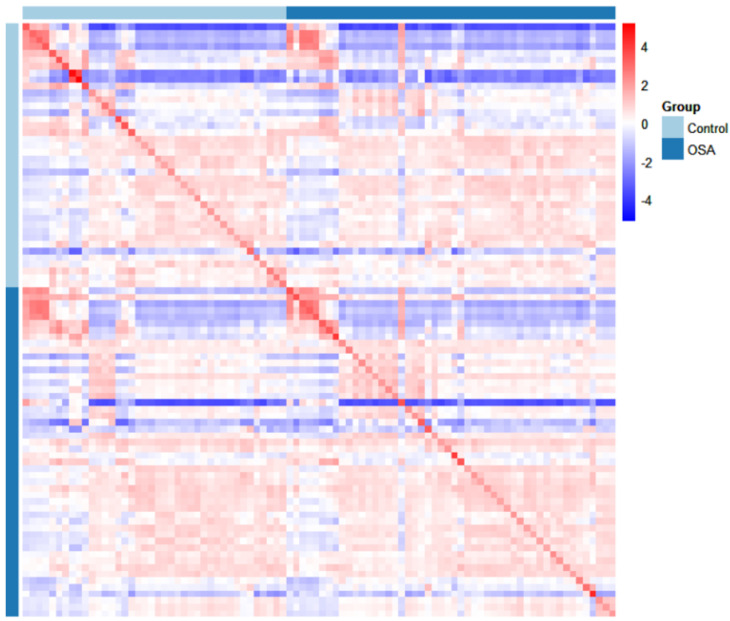
Correlation map for OSA vs. control.

A correlation map (Figure 3) was generated to examine the relationships between each participant’s VOC profiles. The *X*- and *Y*-axes of the heatmap represent the individual participants, with correlations between the participants’ VOC concentrations visualized in the matrix. The map visualizes the correlation between the valid VOC concentrations measured for both OSA patients and controls. In this heatmap, red shades represent positive correlations, while blue shades indicate negative correlations. Strong correlations between certain participants can be observed along the diagonal, with varying degrees of correlation scattered across the matrix. The patterns in the map suggest that there are distinct differences in VOC profiles between OSA patients and control subjects. This visual representation aids in identifying clusters of participants whose VOC signatures may reflect common metabolic alterations associated with OSA, supporting further investigation into specific VOCs as potential biomarkers for disease severity and diagnosis. These patterns suggest that there are consistent metabolic differences between the two groups, offering insights into VOCs that may serve as potential biomarkers for NPC diagnosis.

#### 3.2.5. Correlation Between VOCs and AHI Score

**Figure 4:**
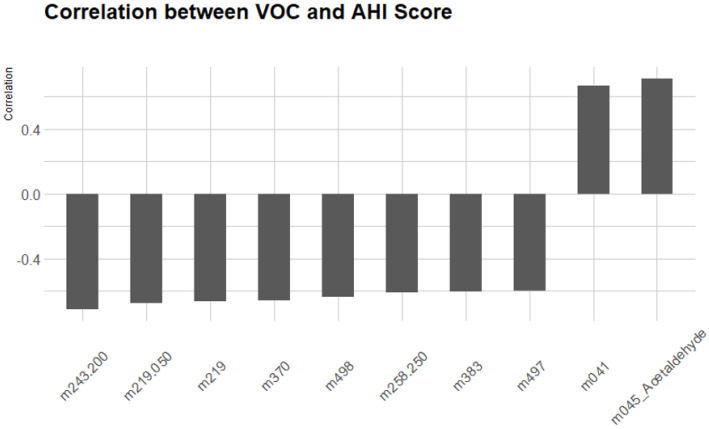
Bar plot of VOC concentrations versus AHI scores in OSA patients. Significant positive correlations are observed.

**Figure 5:**
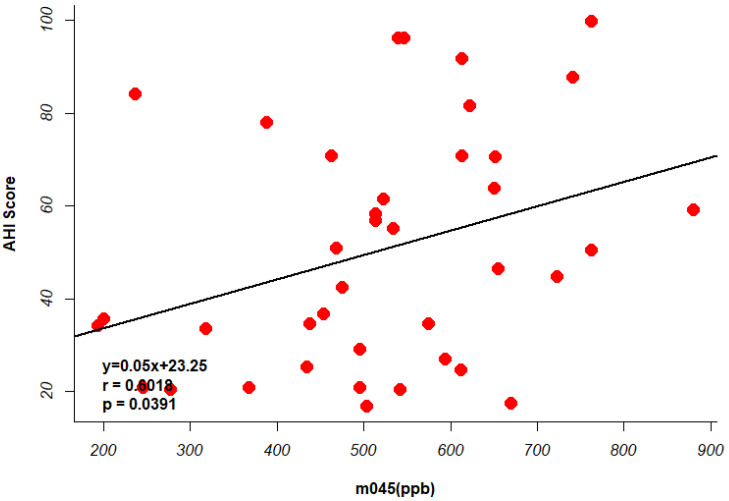
Regression analysis showing the relationship between m045 and AHI scores, indicating a strong association. Each red dot in the figure represents an OSA patient.

**Figure 6:**
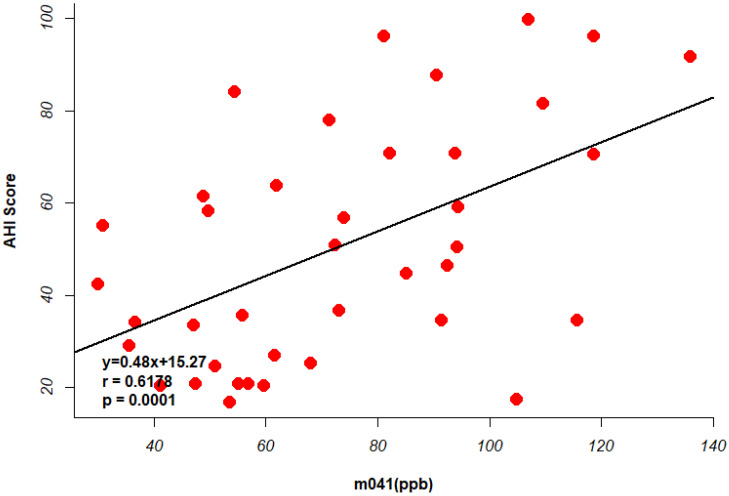
Regression analysis showing the relationship between m041 and AHI scores, indicating a strong association. Each red dot in the figure represents an OSA patient.

Two Figure 4 illustrate the relationship between the identified VOCs and AHI scores in OSA patients. Figure 5 shows the scatter plot of the identified biomarkers’ concentrations against AHI scores, highlighting a significant positive correlation for markers such as m045. Figure 6 presents a regression analysis, further confirming the strong association between these VOCs and the severity of OSA as measured by the AHI.

## 4. Discussion

The results of this study underscore the potential of proton-transfer-reaction mass spectrometry (PTR-MS) in identifying volatile organic compound (VOC) biomarkers that are potentially altered in patients with Obstructive Sleep Apnea (OSA). Significant differences in VOC profiles between OSA patients and controls were observed, identifying specific biomarkers that can distinguish between people with and without OSA.

A key contribution of this research is its novel application of PTR-MS for the non-invasive detection and screening of OSA. The identification of significant VOCs, such as m095.950 and m097.071 for OSA, highlights the sensitivity and specificity of this method [30].

Here, it is important to clarify the potential identities of the *m*/*z* values detected by PTR-MS and their relevance to Obstructive Sleep Apnea (OSA). The *m*/*z* 045, 095.950, and 097.071 ions are significant, and understanding their chemical identity is crucial to interpreting their biological roles.

Firstly, m045 (with an *m*/*z* of 45) cannot be attributed to carbon dioxide since it has a lower proton affinity than water and therefore cannot be ionized by PTR-MS [31]. Instead, acetaldehyde is a more plausible candidate for m045 due to its molecular weight of approximately 44 Da and its established role as a VOC commonly produced during oxidative stress and inflammation. Acetaldehyde is primarily known as a byproduct of alcohol metabolism, and it may also be generated through other metabolic processes, such as those associated with lipid peroxidation, although direct evidence for its production through lipid peroxidation is lacking.

Given that OSA is characterized by chronic intermittent hypoxia, acetaldehyde may be elevated in OSA patients due to increased oxidative stress [32]. Intermittent hypoxia can promote the generation of reactive oxygen species (ROS), which contribute to oxidative stress and inflammation. Insufficient sleep and intermittent hypoxia have also been shown to enhance the expression of genes related to oxidative stress and immune responses, further contributing to the altered metabolic profiles in OSA patients. This oxidative stress may indirectly influence the production of VOCs such as acetaldehyde.

For m095.950 and m097.071, we currently do not have conclusive evidence to identify these compounds with certainty. It is possible that m095.950 corresponds to dichloroethylene, and m097.071 could be linked to 2,4-hexadienal, 1,3-dimethylpyrazole, or cycloheptene. However, further research is required to definitively identify these compounds and to understand their specific roles in the metabolic pathways associated with OSA. More investigation is necessary to clarify their exact origins and implications for OSA pathophysiology.

This study also enhances the understanding of the metabolic changes associated with OSA. The correlation analyses reveal potential metabolic pathways involved in OSA, providing insights for future research and therapeutic strategies. For instance, the strong correlation between certain VOCs and the AHI suggests that these compounds could serve as biomarkers for monitoring OSA severity and treatment efficacy.

Improvements over previous studies include a comprehensive analysis of multiple VOCs and the application of advanced statistical techniques to validate the findings. PLS-DA, heatmaps, and correlation maps provide a robust framework for visualizing and interpreting the data, enhancing the reliability of the results.

While traditional diagnostic methods for OSA, such as polysomnography, are still necessary to confirm the diagnosis, they are often time-consuming, costly, and associated with long wait times. In many healthcare settings, the diagnosis of OSA is often delayed due to long wait times for polysomnography [33]. The advent of portable devices [34] and home-based sleep studies [35] has significantly reduced these delays, yet concerns persist regarding the diagnostic accuracy of these methods, particularly given their cost and the potential need for a repeat Level 1 polysomnography in cases with borderline AHI results [36,37]. Therefore, an improved and more robust screening tool, in addition to existing questionnaires, may be key to optimizing resource allocation and improving patient outcomes. The analysis of volatile organic compounds (VOCs) using PTR-MS could be the solution, offering a rapid, non-invasive, and cost-effective adjunct that could be easily integrated into current clinical settings.

**Figure 7:**
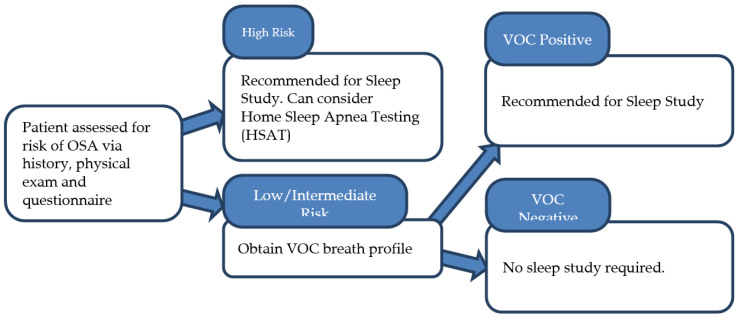
Workflow for breath test validation in low-risk OSA patients, guiding sleep study recommendations based on clinical assessment and VOC profile.

Our study suggests that VOCs could be valuable in identifying patients with severe, markedly elevated AHI scores, potentially allowing for patient stratification and prioritization for earlier in-hospital Level 1 polysomnography, while others may be adequately assessed using HSAT. A proposed workflow for assessing patients for sleep apnea is shown in Figure 7. For patients deemed high-risk based on history, physical exam, and questionnaires, a sleep study will be recommended, and clinicians can strongly encourage consideration of a home-based sleep study given the high pretest probability of sleep apnea. For patients with low to intermediate risk factors, the workflow suggests obtaining the VOC breath profile. If the breath profile indicates significant VOCs, a formal sleep study will be recommended; otherwise, no further action will be recommended.

**Figure 8:**
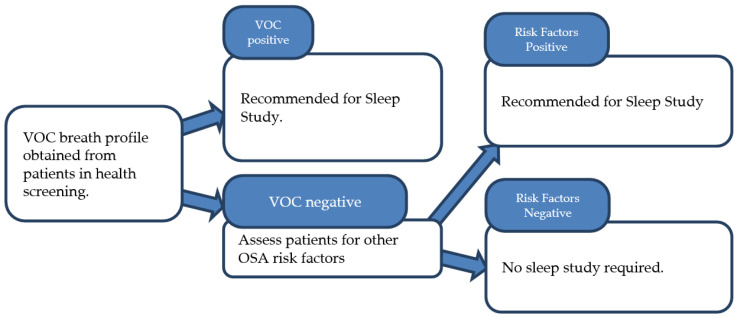
Workflow for OSA health screening using VOC breath test, with additional risk factor assessment for VOC-negative patients.

A second proposed workflow for assessing patients for sleep apnea in the context of health screening is shown in Figure 8. The VOC breath profile can be obtained from asymptomatic patients as part of their routine health screening. If the profile is positive, patients can be strongly encouraged to seek medical consultation for a sleep study. If negative, patients should be further assessed for sleep apnea risk factors, similar to the workflow outlined above, and a sleep study should be recommended for those with significant risk factors. Our study team believes this novel approach would be especially beneficial in a health screening setting, given the low awareness of OSA in many societies [38]. A positive VOC finding in a patient’s breath profile could have a strong impact, motivating them to pursue a formal sleep study, much like how patients with a positive Fecal Occult Blood Test are more likely to undergo a colonoscopy [39].

The integration of VOC biomarkers into OSA diagnostic workflows has the potential to overcome several limitations of existing tools, particularly in terms of accessibility and early detection. Traditional diagnostic methods like polysomnography and HSAT rely heavily on the presence of physical symptoms or clinical thresholds, often leading to delays in diagnosis. VOC analysis, by contrast, offers a non-invasive method to detect biochemical markers that reflect underlying metabolic disturbances associated with OSA, potentially identifying high-risk patients earlier in the diagnostic process. This could improve patient stratification, allowing clinicians to prioritize individuals who may benefit most from an in-depth sleep study, while also identifying those who may not require immediate intervention. By providing an objective, rapid, and cost-effective means of detection, VOC profiling could serve as an effective screening tool, bridging the gap between initial risk assessments and definitive diagnoses and ultimately optimizing both patient outcomes and resource allocation in the management of sleep apnea.

However, several limitations need to be addressed for the clinical translation of PTR-MS-based breath analysis. First, the high cost and complex operational requirements of PTR-MS limit its accessibility and widespread adoption in routine clinical settings. Second, while PTR-MS shows promise, its diagnostic accuracy may not yet rival that of traditional gold-standard methods, making it necessary to use it in conjunction with other diagnostic techniques. Third, the potential confounding effect of age differences between participants in this study has not been fully addressed. Since age can influence metabolic processes and VOC profiles, future studies should consider matching participant groups for age or statistically adjusting for its effects to improve the reliability of findings.

Additionally, the data generated by PTR-MS are sensitive to noise from factors such as diet, race, smoking status, and gender, which could influence VOC profiles and reduce the specificity of OSA biomarkers. Further research is required to mitigate these influences and validate the VOCs identified across larger and more diverse populations [40]. The relatively small sample size in this study is another limitation, potentially affecting the generalizability of the findings. Expanding the sample size in future studies will be crucial for confirming the robustness of the identified biomarkers and for assessing their performance across a broader population.

Lastly, future studies should focus on integrating VOC analysis with other diagnostic modalities to enhance diagnostic accuracy. It is also important to investigate the temporal stability of these biomarkers and their response to treatment interventions, which could provide valuable insights into disease progression and treatment efficacy [27]. Addressing these limitations will be essential for advancing the clinical utility of PTR-MS-based breath analysis.

Moreover, understanding the biological origins of these VOCs and their association with specific metabolic pathways could open new avenues for research and therapeutic intervention. This could include studying the impact of diet, medication, and environmental factors on VOC profiles to further refine the diagnostic capabilities of PTR-MS.

## 5. Conclusions

This study demonstrates that proton-transfer-reaction mass spectrometry (PTR-MS) can effectively identify volatile organic compounds (VOCs) for the non-invasive detection of Obstructive Sleep Apnea (OSA). Significant VOCs, such as m045, m095.950, and m097.071, were identified, showing clear differences between OSA patients and controls. Notably, m045, corresponding to acetaldehyde, emerged as a potential biomarker associated with the AHI, highlighting its relevance in assessing the severity of OSA. The use of advanced statistical methods validated the robustness of these biomarkers, supporting the feasibility of PTR-MS breath analysis in clinical diagnostics. Further validation in diverse populations and integration with other diagnostic tools are necessary to establish clinical utility and improve patient outcomes through early intervention.

## Figures and Tables

**Table 1:** Technical specifications of PTR-MS.

Mass resolution	>1500 m/Δm (FWHM) for *m*/*z* > 79
Response time	<100 ms
Sensitivity	>200 cps/ppbv for *m*/*z* 181
Detection limits	<10 pptv for *m*/*z* 181 (averaged over 1 min)
Linearity range	10 pptv–1 ppmv
Adjustable flow	50–800 sccm

**Table 2:** Study population.

	OSA	Control
Number	49	40
Age (mean ± SD)	52.61 ± 11.36	43.38 ± 12.70
Gender M/F (%)	33/16 (67.3%/32.7%)	24/16 (60.0%/40.0%)
AHI (mean ± SD)	50.98 ± 26.05	6.68 ± 3.96

**Table 3:** Biomarkers identified for OSA with their corresponding *p*-values.

Biomarker (*m*/*z*)	*p*-Value	Compound
m063	0.042	Ammonium formate
m095	0.001	Trifluoro acetonitrile
m096	0.041	dichloroethylene
m097	0.011	2,4-hexadienal, 1,3-dimethylpyrazole, or cycloheptene
m224	0.045	-
m225	0.047	N-hexadecane
m355	0.025	-
m356	0.028	-
m357	0.028	-
m371	0.048	-
m372	0.047	-
m375	0.048	-

## Data Availability

Data are contained within the article.
